# Towards reproducible computational drug discovery

**DOI:** 10.1186/s13321-020-0408-x

**Published:** 2020-01-28

**Authors:** Nalini Schaduangrat, Samuel Lampa, Saw Simeon, Matthew Paul Gleeson, Ola Spjuth, Chanin Nantasenamat

**Affiliations:** 1 Center of Data Mining and Biomedical Informatics, Faculty of Medical Technology, Mahidol University, 10700 Bangkok, Thailand; 2 Department of Pharmaceutical Biosciences, Uppsala University, 751 24 Uppsala, Sweden; 3 Interdisciplinary Graduate Program in Bioscience, Faculty of Science, Kasetsart University, 10900 Bangkok, Thailand; 4 Department of Biomedical Engineering, Faculty of Engineering, King Mongkut’s Institute of Technology Ladkrabang, 10520 Bangkok, Thailand

**Keywords:** Reproducibility, Reproducible research, Drug discovery, Drug design, Open science, Open data, Data sharing, Data science, Bioinformatics, Cheminformatics

## Abstract

The reproducibility of experiments has been a long standing impediment for further scientific progress. Computational methods have been instrumental in drug discovery efforts owing to its multifaceted utilization for data collection, pre-processing, analysis and inference. This article provides an in-depth coverage on the reproducibility of computational drug discovery. This review explores the following topics: (1) the current state-of-the-art on reproducible research, (2) research documentation (e.g. electronic laboratory notebook, Jupyter notebook, etc.), (3) science of reproducible research (i.e. comparison and contrast with related concepts as replicability, reusability and reliability), (4) model development in computational drug discovery, (5) computational issues on model development and deployment, (6) use case scenarios for streamlining the computational drug discovery protocol. In computational disciplines, it has become common practice to share data and programming codes used for numerical calculations as to not only facilitate reproducibility, but also to foster collaborations (i.e. to drive the project further by introducing new ideas, growing the data, augmenting the code, etc.). It is therefore inevitable that the field of computational drug design would adopt an open approach towards the collection, curation and sharing of data/code.

## Introduction

**Fig. 1:**
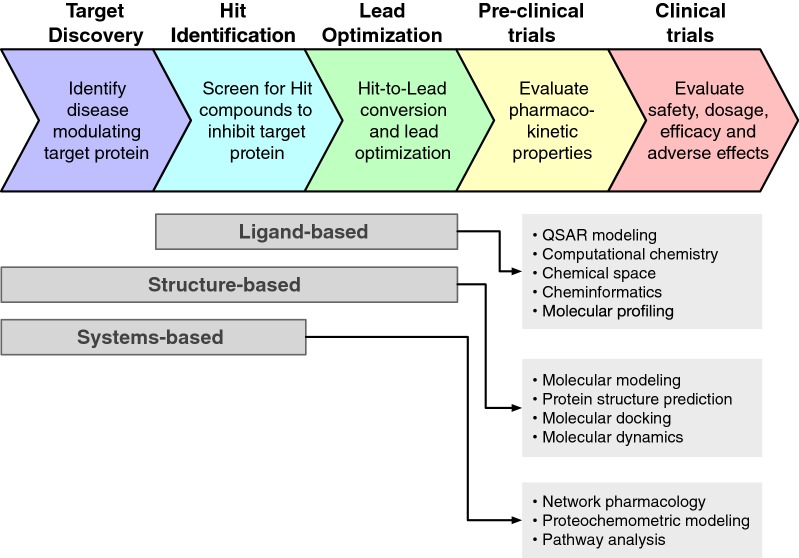
Schematic summary of the drug discovery process overlayed with corresponding computational approaches

**Fig. 2:**
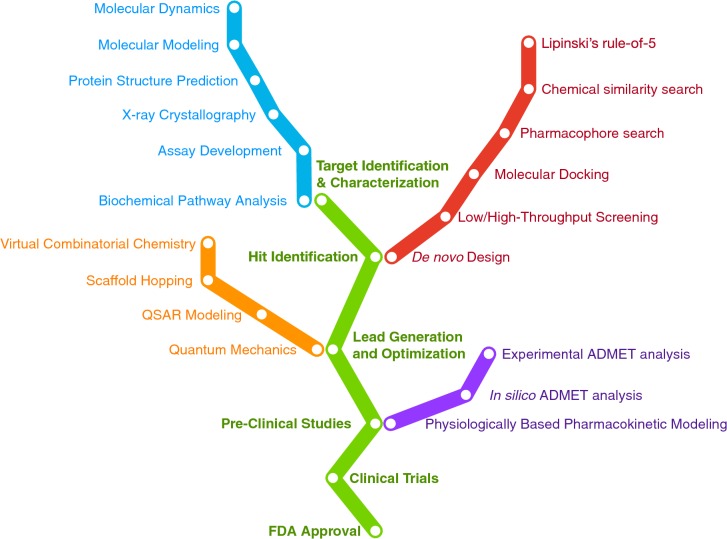
Conceptual map on the experimental and computational methodologies as applied to the drug discovery process [283]. The ordering of terminologies on each of the colored tracks are not of any specific order

Traditional drug discovery and development is well known to be time consuming and cost-intensive encompassing an average of 10 to 15 years until it is ready to reach the market with an estimated cost of 58.8 billion USD as of 2015 [1]. These numbers are a dramatic 10% increase from previous years for both biotechnology and pharmaceutical companies. Of the library of 10,000 screened chemical compounds, only 250 or so will move on to further clinical testings. In addition, those that are tested in humans typically do not exceed more than 10 compounds [2]. Furthermore, from a study conducted during 1995 to 2007 by the Tufts Center for the Study of Drug Development revealed that out of all the drugs that make it to Phase I of clinical trials, only 11.83% were eventually approved for market [3]. In addition, during 2006 to 2015, the success rate of those drugs undergoing clinical trials was only 9.6% [4]. The exacerbated cost and high failure rate of this traditional path of drug discovery and development has prompted the need for the use of computer-aided drug discovery (CADD) which encompasses ligand-based, structure-based and systems-based drug design (Fig. 1). Moreover, the major side effects of drugs resulting in severe toxicity evokes the screening of ADMET (adsorption, distribution, metabolism, excretion and toxicity) properties at the early stage of drug development in order to increase the success rate as well as reduce time in screening candidates [5]. The process of CADD begins with the identification of target or hit compound using wet-lab experiments and subsequently via high-throughput screening (HTS). In particular, the typical role of CADD is to screen a library of compounds against the target of interest thereby narrowing the candidates to a few smaller clusters [6]. However, owing to the high requirement of resources for CADD coupled with its extensive costs, opens the door for virtual screening methods such as molecular docking where the known target of interest is screened against a virtual library of compounds. Although this method is highly effective, a crystal structure of the target of interest remains the main criteria required of this approach in generating an in silico binding model. However, in the absence of a crystal structure, homology modeling or de novo prediction models can still be obtained against the large library of compounds to acquire compounds with good binding affinity to the target [7] which are identified as hits and could be further developed as lead compounds [8]. A conceptual map on the experimental and computational methodologies as applied to the drug discovery process is summarized in Fig. 2.

In recent years, the expansion of data repositories including those with chemical and pharmacological data sets, has significantly increased the availability of large-scale open data for drug discovery. In addition, more data are being deposited into these domains on a daily basis, with some repositories containing tens of millions of compounds (e.g. PubChem and ZINC databases) [9]. The availability of such large-scale data sets has had a significant impact on the drug discovery process. Moreover, this process may help address many of the unmet needs in drug discovery and design such that the access to these data may help with the rapid identification of compounds to validate targets or profile diseases which will further encourage the development of new tools and predictive algorithms. Furthermore, large bioactivity data sets can be used for the identification of quantitative structure–activity relationships (QSAR) or classification models, allowing prediction of compound activities from their structures. Such predictions can contribute to molecular target elucidation, drug ADMET prediction and potential drug repurposing [10]. However, with all the predictive methods, the quality and relevance of the data acquired are paramount in determining the accuracy and applicability of the resulting models. Therefore, as data sets become more readily available due to the open science initiative, the emphasis has now moved towards quality, rather than the quantity of raw data. Indeed, many analyses have been published assessing the quality of screening libraries that identify compounds responsible for many of the false-positive results [11, 12] or investigate compound structure accuracy in various repositories [13, 14]. Hence, any progress made within just this one area will have a profound impact on improving the development of novel and safe drugs. Nevertheless, with the increasingly rapid growth of these public data sources therefore efforts in ensuring the quality and interoperability will be essential for maximizing the utilization of data.

In the midst of big data expansion (i.e. borne from omics data) that are available for computational drug discovery, proper efforts for ensuring the quality of these data are made possible through data curation and pre-processing as carried out by database and repository providers. Workflows and pipelines in the form of markup languages, codes or software tools have become instrumental in ensuring the reproducibility of computational research as it helps to materialize the actual steps and procedures taken during the entire computational study. Discussion on the availability and current efforts undertaken in the field of computational drug discovery (i.e. also encompassing bioinformatics and cheminformatics) in regards to research reproducibility is provided in this review article. During the revision phase of this manuscript submission, an excellent commentary article by Clark [15] addressing the importance of reproducibility in cheminformatics was recently published. Moreover, a blog post by cheminformatic researchers [16] also reaffirmed the significance of this point and the timely manner of the topic of this review article so as to encourage further developments and paradigm shifts in computational drug discovery and neighboring fields (e.g. bioinformatics and cheminformatics) pertaining to research reproducibility.

## Research documentation

Scientific experiments have long preceded digital logging of laboratory activities. Documentation of experimental results has traditionally been kept within the confinement of paper-based notebooks whereby the scientific benefits of which is to allow subsequent reproduction of the documented experiment, while its legal use is to serve as a proof of inventorship [17]. The reporting of science is fundamental to the scientific process, which, if done clearly and accurately, can help advance knowledge and its reproducibility [18]. All professionals working in life sciences are familiar with the importance of keeping laboratory notebooks. Although, science as a field has advanced over the centuries, the methods of recording data (i.e. in a paper-based, inked and bound notebook) has remained unchanged. In addition, the current reproducibility crisis has put the spotlight on data recording. Therefore, unsurprisingly, many industries and laboratories are now shifting to a digital form of record keeping, the electronic laboratory notebooks (eLNs) [19].

eLNs have been introduced as a digital alternative to the paper-based version but with enhanced capabilities such as search capability, integration with instrumentation, etc. [20]. Scientists are increasingly adopting the use of eLNs in their research laboratories owing to the inherent need to organize the growing volume of biological data [21]. Recently, Schnell [22] had proposed ten simple rules for a computational biologist’s laboratory notebook, which highlights the importance of documenting all the minute details that were carried during the course of project from start to finish (i.e. applicable to all scientific disciplines) while also making use of version control, virtual environments and containers (i.e. applicable to computational disciplines). Particularly, which software version was used, which parameter values were used, which specific algorithms and specific options were utilized for the calculation, etc. Moreover, scientists are making these notebooks publicly available so as to support the open science initiative (i.e. also termed “open notebook science”) [23, 24] and in doing so foster the sharing of unpublished experimental data and analysis (i.e. known as “dark data”). These interactive notebooks (i.e. also known as iPython/Jupyter notebooks) have evolved to the point that it is possible for the code used to perform the data analysis to be shown alongside the explanatory text and visualizations (e.g. images, plots, etc.), thereby affording easy comprehension of the experimental results and its underlying code, thus facilitating reproducible research.

The iPython notebook was created in 2001 by Fernando Perez and has since evolved to the more general and powerful Jupyter notebook [25] with support for more than 40 programming languages (e.g. Python, R, Javascript, Latex, etc.). For the sake of data sharing, it is common practice to store the Jupyter notebooks (i.e. used hereon to also refer to the iPython notebook) on GitHub (i.e. or other web repository such as BitBucket). Such notebook files can then be rendered as static HTML via the nbviewer [26]. Recently, GitHub also made it possible for Jupyter notebook files to render directly on its repositories. Owing to the static nature of the rendered notebook the resulting HTML is consequently not interactive and therefore not amenable to modifications. A first step towards solving this limitation is made by the Freeman lab at Janelia Research Campus in their development of binder [27], a web service that converts Jupyter notebook files hosted on GitHub to executable and interactive notebooks. Google CoLaboratory [28] is another interface which utilizes the Jupyter notebook environment for the dissemination of research and education. Google Colaboratory is a free platform whereby projects can be run completely on the cloud, without the need for any software setups while the *“notes”* are stored entirely on Google Drive and can be easily accessed and shared.

At the other end of the spectrum are cloud-based word processors such as Google Docs, Overleaf, ShareLatex and Authorea that facilitate collaborative writing of experimental findings and results in the form of manuscripts, books and reports. A distinctive feature of these applications is the possibility for several users (i.e. who can be physically located in different parts of the world) to be able to work on the same document at the same time. Most of these web applications serve as only word processors that house the text of a manuscript but does not allow integration with the Jupyter notebook. In fact, only Authorea integrates interactive Jupyter notebooks (i.e. also hosted by Authorea) into their application so that users can play around with the parameters and come up with customized figures and plots.

## Science of reproducible research

### Reproducibility crisis

According to an online survey conducted by Nature of 1576 researchers, it was revealed that 52% of researchers agreed that there is a significant reproducibility crisis while 38% agreed that there is a slight crisis. On the other hand, 3% of those surveyed do not think that there is such a reproducibility crisis while 7% of researchers are not aware of its very existence [29]. These results suggests confusing viewpoints as to what constitutes reproducible research. In addition, when asked to identify the problem associated with this crisis, the same survey reported over 60% of respondents believe that the pressure to publish and selective reporting contributed to the problem. Furthermore, lesser contributing factors reported were unable to replicate the work in the lab, low statistical power and obstacles such as reagent variability or the use of specific techniques that are difficult to replicate.

The concept of reproducibility in science depends on the dissemination of knowledge and the reproducibility of results. To facilitate this, the accurate and clear reporting of science should be a fundamental part of the scientific process. Plavén-Sigray et al. [18] believe that the readability of a scientific research is one of the main factors for reproducible and accessible literature. From a compilation of 709,577 abstracts from 123 scientific journals published between 1881 and 2015 on biomedical and life sciences coupled with readability formulas, the authors concluded that the readability of scientific literature has been decreasing over time. Lower readability could in turn discourage accessibility, particularly from non-specialists and the importance of comprehensive texts in regards to the reproducibility crisis cannot be ignored.

Another aspect of the reproducibility crisis can be seen during the data analysis whereby it can be difficult for researchers to recognize *p*-hacking also known as data dredging [30] (i.e. the phenomenon where researchers select statistical analysis which portray insignificant data as significant) due to confirmation and hindsight biases which encourage the acceptance of preconceived outcomes that fit expectations [31]. Hence, there is an increased concern that most published articles are based on false or biased results [32]. In addition, several studies have pointed out that the high rate of non-replicable discoveries is a consequence of basing conclusive findings on a single study assessed via only the statistical significance (i.e. the *p*-value) [32–34]. Therefore, in order to combat this disturbing trend, striving towards the FAIR (Findable, Accessible, Interoperable and Reproducible) [35] principle in research practices can help to ensure that models and studies are FAIR for them to be consumed and integrated on-demand. Hence, studies using open data derived from analysis according to the FAIR principles, will pave the way towards iteratively better science with higher confidence in the reproducibility of research [36].

### Reproducibility versus replicability

It is important to note that the terminology found across the scientific literature such as reproducibility, replicability, reusability, recomputibility and their associated definitions are not standardized and thus has led to confusion regarding their usage. *“Reproducibility”* has been defined in the dictionary as *“the ability to produce, form or bring about again, when repeated”* [37]. In the context of computational research, the term *“reproducible research”* was first coined by Jon Claerbout in 1990, the geophysicist who implemented the standard for maintaining and building executable programs from the source code leading to the construction of computational results known as the Stanford Exploration Project in published articles [38]. An important issue for reviewers and authors alike, reproducibility acts as a bedrock principle for the validation in experimental scientific research. However, with such emphasis placed on reproducibility in experimental sciences, two conspicuous discrepancies were highlighted by Casadevall and Fang [39]. First, while the work conducted and published by scientists are expected to be reproducible, most scientists do not partake in replicating published experiments or even read about them. Furthermore, despite the obvious prerequisite in most reputable journals whereby, all methods must be reported in adequate detail so as to allow replication, no manuscripts highlighting replicated findings without the discovery of something novel are published. Thus, the reproducibility of any given published research is assumed, yet only rarely is that notion tested. In actuality, the reproducibility of experiments are only highlighted when a given work is called into question [40]. Hence, the consistency of this basic supposition relies heavily on integrity of the authors publishing the results and the trust afforded to them by the publishers and readers [39]. Ironically, suspicions of data falsification are sometimes heightened when results are deemed as *“too good to be true”* [40]. Therefore, this replication debate provides an opportunity to redefine the differences between replicability and reproducibility.

As such, strict definitions of both terms are also available and could be useful in discerning slight differences that occur by either repeating or reproducing an experiment/workflow. According to the *Guide to the expression of uncertainty in measurement* [41], reproducibility is defined as the *“closeness of the agreement between the results of measurements of the same measure and carried out under changed conditions of measurement”* while repeatability or replicability is defined as the *“closeness of the agreement between the results of successive measurements of the same measure and carried out under the same conditions of measurement”*. Although the mismatch of both terms is not so critical in some cases, it is important to clarify the main differences. For example, if the experiment/model conditions are close or identical, they should be successfully repeated (i.e. repeatability or replicability). On the other hand, if the experimental/model conditions are changed to some degree, the exact or close match results may not be obtained but the methodology should be sound (i.e. reproducibility).

### Reusability versus reliability

In life sciences, the reliability of a published protocol is a pressing matter upon implementation. Reusability is more prevalent in computer science in which codes created by an individual or groups of individuals that are shared on public repositories, can be reused by others as well as facilitate future work to be built upon it. Hence, enabling reusability represents an important catalyst that would help to advance the field. Conventionally, scientific research relies on results from independent verification. Specifically, when more people verify an observation or hypothesis, the more trustworthy it becomes. A conjecture, on the other hand, without verification is therefore not considered to be well-thought-out. Thus, replication represents an important facet of verification within which theories are confirmed by equating predictions in relation to reality. For computational research however, no established verification practices exist as of yet [42]. Although a research may be reproducible, the quality, accuracy or validity of the published results are not guaranteed. Therefore, simply bringing the notion of reproducibility to the forefront and making it as routine as keeping a laboratory notebook, would help set the stage for a reproducible atmosphere. Encouragingly, the minimum information checklist brought together under the umbrella of the Minimum Information for Biological and Biomedical Investigations (MIBBI) project [43] has helped to ensure that all pertinent data is provided by researchers. Furthermore, bioinformatics software typically involve a wide variety of data formats which can make the execution of replicability a little more difficult. However, softwares pertaining to data exchange and analysis such as the Proteomics Standard Initiative for molecular interactions (PSI-MI) for proteomics [44] and the Biological Pathway Exchange (BioPAX) language [45] representing metabolic and signaling pathways, molecular and genetic interactions and gene regulation networks, have been developed to improve this. In addition, the Workflow4Ever project [46] caters to the same aim using a different approach.

The underlying aim of reproducing any given research/experiment is so that the work being proposed can be extended rather than just to confirm it. It also then, makes perfect sense that the extensibility of methods in the computational realm is taken into account during the design phase [47]. Conducting research can, in this day and age, no longer be a lone endeavour; rather, collaborations have permanently made their way into the sciences. In that respect, many bioinformatic tools have been developed under a joint effort where one group extended the work of another group such as the Bioconductor [48] and Galaxy [49–51] projects. In addition, a tool specifically made for analyzing phylogenetic data, Beast 2 [52] and Beast 2.5 [53], emphasizes modular programming techniques into its software in order to allow the software to be extensible by users. Furthermore, the Jupyter Notebook [25] offers a dynamically updating, error-correcting tool for the publication of scientific work, thus facilitating extensibility. In addition, protocols.io [54] is an open access repository for scientific protocols that allow lab members to write and edit collaboratively.

This debate further behooved the question as to who would benefit from the detailed accumulation of methods in scientific papers or codes shared on various virtual platforms. Perhaps, it would be most advantageous for the new scientist as they can learn to use novel software/protocol without going into too much detail and without having to write the code themselves. In addition, it allows the general public to make use of, and maneuver a minimal working environment while saving time which could possibly provide a fresh perspective to existing research data.

### Open Science

In the last decade or so, the sharing of scientific data has been promoted by a growing number of government and funding agencies [55, 56]. As such, open access to data from research networks, governments, and other publicly funded agencies has also been on the rise given the policies that promote them [57]. However, the sharing of data in terms of policies varies dramatically by field of research, country, and agency, yet many of their goals are conjoint. Upon analysis of these policies, Borgman [58] found that the data sharing policies are based on four main features (i.e. reproducible research, making data available to the public, influencing investments in research, and advancing research and innovation). Epistemically, the impulse for the production of new knowledge with the reuse of data through open sources, is the key take away from these arguments [35, 59]. The proposed benefits of sharing can only be accomplished if and when the data is shared and/or reused by others [58]. Hence, *“data sharing”* refers to the idea and implementation of data release and in its simplest form, is the act of making data readily and easily available and accessible [60]. Data sharing thus, encompasses many means of releasing data, while saying little about the usability of those data. Some ways whereby researchers share their data are private exchanges, posting data sets on websites (e.g. GitHub or Figshare); depositing data sets in archives or repositories (e.g. PubChem or ChEMBL); and supplementary materials provided in research articles [61]. Data papers represent a newer avenue in the research field whereby descriptions similar to the *“Methods”* section of a traditional research article are published with greater details regarding the processes used for data collection, experimentation and verification [62, 63].

Furthermore, reproducibility can be seen to critically affect various aspects of research, especially in the field of science [29]. However, these days bioinformatics plays a distinct role in many biological and medical studies [64]. Thus, a great effort must be made to make computational research reproducible. As such, many reproducibility issues that arise in bioinformatics may be due to various reasons such as version of bioinformatics software, complexity of its pipeline and workflow, technical barriers ranging from insufficient data to hardware incompatibility, etc. [65]. This crisis has been described by Kim et al. [66] whereby the authors compare the hidden reproducibility issues to an iceberg which is only noticed at a fraction of its actual size, highlighting the significant gap between the apparent executable work (i.e. portion of iceberg that can be seen above water) and the necessary effort required to practice (i.e. the full iceberg).

To deal with this reproducibility crisis, Sandve et al. [67] proposed ten simple rules for reproducible computational research, through which the authors encourage researchers to responsibly and consciously make small changes during their computational workflow in order to achieve reproducibility habits that benefit not only the researchers but their peers and the scientific community on the whole. In our humble opinion, one of the most important point from the article stressed the importance of publicly sharing the data and source code so as to foster reproducibility of the work and in turn move science forward. One of the projects that implemented most rules laid out by Sandve et al. is the Bioconductor project [48] which is an open software that encourages collaborations in the fields of computational biology and bioinformatics. In addition, BaseSpace [68] and Galaxy [51] represent examples of both commercial and open-source solutions, that partially fulfill the ten simple rules laid out in the aforementioned review. However, workflow customizations on such environments are not implementable, for example, BaseSpace have strict application submission rules and being cloud based, have to cope with ethical and legal issues [69].

The applications and pipelines in bioinformatics require a substantial effort to configure, therefore container-based platforms, such as Docker [70], have emerged to allow the deployment of individual applications that have an isolated environment for the installation and execution of a specific software, without affecting other parts of the system. In this regard, many docker-based platforms have been produced such as BioContainer [71], a community-driven, open-source project based on the Docker container that can be easily accessed via GitHub; Bio-Docklets [72], a bioinformatics pipeline for next generation sequencing (NGS) data analysis; and Dugong [73], a Ubuntu-based docker that automates the installation of bioinformatics tools along with their libraries and dependencies on alternate computational environments. The above-mentioned platforms utilize the Jupyter Notebook as an integration platform for delivery and exchange of consistent and reproducible protocols and results across laboratories, assisting in the development of open-science. In addition, the Reproducible Bioinformatics Project [74] is a platform that distributes docker-based applications under the framework of reproducibility as proposed by Sandve et al. Furthermore, the more recently established Human Cell Atlas [75] is an ambitious project encompassing more than 130 biologists, computational scientists, technologists and clinicians. Their aim is to help researchers answer questions pertaining to the human body in diverse biological fields. However, to provide maximum impact and continued collaborations, the project will be a part of open science on multiple levels to ensure that the results are of high quality and are technically reproducible. The initiative currently includes members from 5 continents and more than 18 countries, including Japan, Israel, South Africa, China, India, Singapore, Canada and Australia. The work conducted by this initiative in a large-scale international, collaborative and open effort may bring different expertise to the problems and could dramatically revolutionize the way we see our cells, tissues and organs.

### Computational reproducibility ecosystem

So the question is, how does one go about making their own research reproducible? For a computational life scientist there are a plethora of resources that are enabling factors for data-driven research and it is the intent of this section to attempt to provide a broad if not extensive coverage. Conceptually, a reproducible ecosystem could be thought of as environments or enabling factors that, on one end, allow the practitioner to archive and share their data and codes while on the other end, allow third-party users to gain access to these resources so that they can build upon them in their own independent projects. A more in-depth coverage of this topic is provided elsewhere [76, 77]. Traditionally, data (and rarely codes) accompanying a research article containing computational component(s) are provided in the Supplementary. In recent years, several web-based services are available as enabling proponents for computational reproducibility as will be discussed hereafter.

Data repository is a relatively general term used to reference a storage site specifically delegated for data depositions. As part of the reproducibility era, many appropriate public data depositories are now available such that, authors are able to deposit their raw research data into discipline specific and community-recognized repositories (i.e. GenBank, PDB, PubChem, etc. for discipline specific and figshare, Dryad digital repository, Zenodo, Open science framework, etc. for general repositories) [78]. The Dryad digital repository [79] is an open, curated, reusable and easily citable resource for scientific data. In addition, Dryad was initiated as a non-profit organization employing the joint data archiving policy (JDAP) for journal submissions with integrations. Moreover, DryadLab [80], a project of the Dryad digital repository, represents an open-licensed educational module which has been developed by collaborations with researchers and educators for students of all levels (e.g. secondary, undergraduate and graduate) to make use of real data in their work. Furthermore, figshare [81], a website conceived by Mark Hehnel, is a platform whereby scientists can deposit all of their data which, when uploaded, is given a citable digital object identifier (DOI) based on the Handle System thereby ensuring efficient searches and security of the stored data for long-term access. Therefore, the massive amount of data accumulated through scientific research activities that never get published, can be shared. This in turn could drastically reduce the expenses involved with the attempt to duplicate experiments [82]. Moreover, Figshare also encourages the deposition of data that has been generated but never published. Similarly, Zenodo [83] is an open research repository developed by OpenAIRE and CERN in 2013 based on the Invenio digital library framework which also supports DOI versioning for researchers of all fields. Additionally, the reporting of research funded by the European Commission via OpenAIRE is also integrated into Zenodo whereby all research is stored in the cloud. Furthermore, the Open Science Framework (OSF) [84] is a cloud-based tool promoting the open and centralized management of scientific workflows at all stages of the research process, with integrations from many other data hosting/repository services (e.g. Dropbox, GitHub, Google Drive, figshare, etc.). The OSF was developed in 2013 by a non-profit organization known as the Center for Open Sciences (COS) [85] for conducting research that supports and builds the scientific community by promoting the reproducibility and integrity of research. In addition, OSF not only supports researchers in the scientific community, but also software developers and publishers allowing for institutions to create and manage projects which can be shared via posters and presentations in meetings and conferences [86]. Additionally, publishers are further facilitating data sharing by establishing data journals that allow researchers to share their data in publication format that comes equipped with citable bibliographic details and DOI (i.e. without the need to provide full analysis that is typical of full-length research articles). Notable examples include Nature’s Scientific Data [87], Elsevier’s Data in Brief [88], MDPI’s Data [89] as well as F1000Research [90]. It is also worthy to note that pre-prints also represents an important source for disseminating not only data papers but also full-length research articles while the actual manuscript may be under the peer-review process. Notable pre-print journals include arXiv [91], bioRxiv [92], ChemRxiv [93] and PeerJ Preprints [94].

Code repositories act as an archive for file and web facilities which are deposited either publicly or privately. Most often, they are used by open-source software projects as they allow developers to submit organized patches of code into the repositories supporting version control. Some of the main examples include GitHub and BitBucket. GitHub is a web-based, distributed version control system that allows developers to collaborate with people anywhere in the world, using a single codebase on the GitHub web interface. Both small and large projects can be handled with speed and efficiency using GitHub. Similarly, BitBucket [95] is a Git and Mercurial code management and collaboration platform used by professional teams to build, test and deploy software. A special feature included in BitBucket known as pull requests, allows code review that results in a higher quality of the code produced which can also be shared amongst the team. In addition, branch permissions in the BitBucket software provide access control thereby ensuring that only the right people are able to make changes to the code. It should be noted that not all codes and data can be made publicly available and in such circumstances, private repositories such as GitLab [96] represents a lucrative solution. Furthermore, cloud-hosted source repositories such as Assembla and Google Cloud Platform, provide storage facilities where the code can be kept secure without the threat of a hardware collapse. Assembla [97] represents the only multi-repository provider used for hosting repositories (e.g. Subversion, Git and Perforce) in the cloud that also answers the requirements for compliance. Assembla also provides the use of cross-platform applications which can integrate seamlessly with other modern cloud services such as JIRA, Jenkins and Slack. The Google Cloud Platform [98] on the other hand, utilize Git version control for supporting collaborations of various applications, including those running on the App Engine (i.e. a cloud computing platform for hosting web applications) and the Compute Engine (i.e. the service component of the Google Cloud Platform). Google Cloud also provides a source browser through which, the viewing of uploaded repository files is possible. Moreover, it is able to integrate source code already present on GitHub or BitBucket seamlessly onto its cloud platform.

Interactive code platforms such as Binder and Code Ocean, allow subscribers to collaborate in real-time via a remotely hosted web server without the need for software installations. Binder [27] allow subscribers to deposit their GitHub repositories containing the Jupyter notebook via a URL, which is then used to build a Binder repository. Dependency files from the uploaded environment generates a Docker image of the repository. Furthermore, a JupyterHub server hosts repository contents thereby allowing easy access to live environments as well as facilitate sharing with others using a reusable URL. An article by Sofroniew et al. [99] on neural coding published in eLife made use of Binder to share data on all neural recordings. Furthermore, an article published in Nature by Li et al. [100] on the robustness of neural circuits, used the Binder platform to share their computational simulation results. In addition, Code Ocean [101] is a cloud-based computational platform that allows users to share and run codes online, thus encouraging reproducibility. Partnership between Code Ocean and publishers would further encourage reproducibility in which readers can gain access to the executable algorithms right from the published articles as is the case for IEEE journals [102].

Taken together the aforementioned resources supports the hosting, using and sharing of data/code, which sets the stage for the paper of the future as also discussed by C. Titus Brown in a Nature TechBlog [103]. Brown also suggests that non-technophiles can learn the tools of the trade that facilitates computational reproducibility by attending training workshops such as those provided by Software Carpentry [104] and Data Carpentry [105].

## Model development in computational drug discovery

*In silico* models can be generated to study a wide array of chemical and biochemical phenomenon. In this section, we consider aspects of the model building process and key issues on what is needed to generate sufficiently accurate, reproducible models. We consider the full range of computational models ranging from ligand-based cheminformatic methods and molecular mechanics-based models to high-level structure-based simulations involving protein-ligand docking or protein-substrate reactivity.

In the context of computational drug discovery, it is in our view that it is important to differentiate between the reproducibility and the predictability of a computational model. The former relates to how accurately subsequent predictions on the same compounds change over time when compared to that proposed using the originally developed computational model. Alternatively, reproducibility could also relate to the model building procedure, whether the data was sufficiently sampled such that repeating the process would not lead to dramatically different results. In most cases, differences can occur between different models that depends on how the data sets were sampled and how the models were generated, implemented or maintained. For example, deviations can occur if: (1) an unrepresented data set are selected for model building, (2) following implementation a different variant of a particular descriptor engine is used for the model (i.e. clogP, AlogP, etc.) or (3) during the production phase, descriptor coefficients are truncated or rounded off. These sources of reproducibility errors can of course be easily monitored by periodically re-running models on the original data sets and descriptors. This naturally brings us to the intrinsic accuracy of the model itself, which is related to how well our theoretical description of the phenomenon (i.e. being models) can actually describe the physical event taking place. *In silico* models cannot describe an experimental event with the same level of accuracy than performing the experiment a second time. Thus, small differences in the reproducibility of a given model may not make a significant difference to the utility of the method in the real world. Furthermore, models that have what might be considered to be a finite predictivity ($r^2$ of  0.5) can indeed be useful in drug discovery. In these cases, updates to particular descriptors engines at the back-end of a prediction tool will in all probability have negligible impact on the overall model statistics if generated on a large, diverse data set. Thus, while the absolute predictions for each compounds will change, the overall effect on the accuracy of the prediction is likely to be negligible. A key issue is therefore to build an intelligent system or workflow such that rounding errors or other subtle differences can be differentiated from the more serious algorithmic or implementation errors.

### Chemical and biological data repositories

The implementation of open data initiatives by many fields including bioinformatics and proteomics has dramatically risen in the past few years with the Human Genome Project being paramount in guiding the scientific community towards open science [106]. However, researchers in the pharmaceutical industries lack the appropriate informatics knowledge that would allow them to completely make use of such platforms. Hence, the availability of cheminformatic tools that are easy to use can help reduce time and cost in this complex drug discovery field [107]. Similarly, various connections between protein and ligands can also be established using these widely available resources [108]. The presence of a large number of experimental and biological databases containing relevant screened compounds is easily accessible via a public domain. Among them, the most widely used databases are ChEMBL [109] and PubChem [110]. The ChEMBL bioactivity database is a large open-access drug discovery database comprised of more than 2.2 million freely available compounds obtained from over 1.8 million assays having around 15 million activity values [109]. In a similar fashion, PubChem was established by the National Center for Biotechnology Information (NCBI) as a public repository that gathers information on biological activities of small molecules. In addition, PubChem currently contains a database of about 96.5 million compounds with bioactivities for greater than 237 million [110].

Furthermore, another publicly accessible database known as Binding Database or simply BindingDB [111] contains experimental small molecules interaction data from patents and scientific articles making up more than 1.4 million protein-small molecule affinities with over 7,000 proteins involved with greater than 650,000 small molecules as of January 2019 [112]. In addition, DrugCentral [113] and DrugBank [114] are comprehensive resources focusing on FDA approved drug that combines the chemical, pharmacological and pharmaceutical information of the drug with the sequence, structure and pathway information of its target. This latest update of DrugBank [115], shows a tremendous increase in drug-drug interaction data for ADMET properties as well as additional new features such as pharmaco-omics data with special focus on pre-clinical and clinical trials. These additions and enhancements are intended to facilitate research in pharmacogenomics, pharmacoproteomics, pharmacotranscriptomics, pharmacometabolomics, pharmacokinetics, pharmacodynamics, pharmaceutics and drug design and discovery. In addition, databases such as CARLSBAD [116], BRENDA [117] and ExCAPE-DB [118] contain uniformly presented data integrated and curated from various repositories .

### Ligand-based approaches

Ligand-based drug design is based on identifying key features that give rise to biological activity and aiming to incorporate, improve or identify new chemotypes with similar characteristics. Pharmacophore-based models are based on 2D or 3D methods and assume that all molecules that contribute to said pharmacophore bind in a similar manner to the prospective target [119–121]. Such similarity may be used to identify compounds with the same or similar features, or can be employed in conjunction with statistical methods to give either structure-activity relationships (SAR) or the more extensive QSAR. Such methods are useful to ascertain trends within primary screening data. The intuition of the medicinal chemist is critical at the beginning of a project, however the large amount of early screening data generated mean that visual analysis of the data is not practical [122]. Thus, compounds clustering and SAR analyzes can provide a simple, efficient means to explore or generate initial SAR. This allows one to identify a series of molecules with the greatest potential and develop new molecules with relatively localized structural changes to significantly assess the activity landscape.

In the context of chemical risk assessment, toxicological profiles of chemicals (cosmetics, industrial chemicals, food chemicals, etc.) are often tested in animals prior to human consumption or usage. QSAR has been proposed as a promising replacement to animal testing [123] or if experimental testing is inevitable then QSAR can help to (1) supplement experimental data and (2) prioritize chemicals for such experiments [124, 125]. Particularly, QSAR can help in regulatory purposes as it can be used to generate structural alerts or *“expert rules”* derived from SAR observations that relates a structural template or functional group to a particular adverse event (i.e. toxicity and undesirable pharmacokinetic properties). As the name suggests, the rules can be a result of expert intuition or from statistical analysis of representative data sets. Many examples exist, including for DNA reactivity [126], toxicity [127, 128], skin sensitivity [129, 130], pan assay interference (PAINs) compounds and general purpose filters for undesirable compounds [131–133]. An alert does not mean that a toxic event is to be expected per se, rather it acts as a qualitative prediction of increased risk. This means that such models can be used for guidance purposes only. Indeed, Alves et al. [134] noted the concern that these structural alerts can disproportionally flag too many chemicals as toxic, which questions their reliability as qualitative markers. The authors state that the simple presence of structural alerts in a chemical, irrespective of the derivation method, should be perceived only as hypotheses of possible toxicological effect.

QSAR modeling involves generating multivariate predictive models using chemically relevant descriptors (e.g. structural counts, fingerprints, 2D and 3D molecular properties, etc.) along with biological activities [135–140]. There are thousands of potential molecular descriptors of numerous types that can be used to explore the complex relationship between structure and response. In such cases, care needs to be taken as the probability of finding spurious correlations, particularly with small data sets, is significant [141, 142]. Biological responses (e.g. inhibitory activity) will typically undergo logarithmic transformation or be used to define sub-classes to facilitate the statistical model building process [143]. Model building can be performed using a wide variety of methods ranging from simple statistical methods (e.g. multiple linear regression) to machine learning methods (e.g. random forest, artificial neural network, support vector machine).

Model performance then needs to be assessed using a wide range of statistics, including correlation coefficients, estimates of prediction error, etc. For classification models, false positive and negative rates as well as holistic measures such as the Kappa statistic or the Matthew’s correlation coefficient are recommended [144, 145]. To understand the true predictive capability of the model it can be instructive to look at how the errors or correlation compare with repeat measurement from the experimental assay being modelled. QSAR models cannot be more predictive than the data they are built on. If such a situation is encounter, it would suggest the model is overfitted and may not extrapolate well to future compounds [143]. In such cases, additionally statistical validation in the form of leave one, or leave many out cross validation, or Y-randomization trials can be useful [142, 145].

Aside from assessing the performance of constructed QSAR models, current efforts are already in place for establishing the reliability of QSAR models, for instance, by using conformal predictions [146, 147] and applicability domain [143], which have been proposed as promising approaches for tackling this issue. Putting this into perspective, the statistical performance as produced by conventional metrics such as $R^2$ or RMSE suggests how well the model is performing on the prediction task but it does not consider whether such predictions are made on compounds falling within the boundaries of the applicability domain or the degree of certainty that the model has on the predicted bioactivity of compounds. Such confidence and the degree at which a compound falls within the applicability domain would greatly assist in compound prioritization. A further practical look into applicability domain will be discussed in the forthcoming paragraphs.

QSAR models can only be as good as the data that they are built on therefore, it is to be expected that they would not be able to predict as good as repeating the experiment a second time. QSAR models are highly useful as a first filter however, users and developers face a number of issues while generating and using the models in practice [148]. The quality of the models can be evaluated with statistical parameters, including correlation coefficient or root mean square error (RMSE). It is expected that the RMSE cannot be smaller than the RMS of the experimental method, otherwise the model is overfitted [149]. Many data sets that require investigation consist of diverse compounds sets of finite sizes (i.e. commonly 100-10000 in size) and are sometimes termed global models [150, 151]. This means that any model built could easily be overfitted due to the typically small number of observations and large number of descriptors and modeling methods. Another issue is that any new compounds may not be very similar to those used to build the model and may therefore be poorly predicted, which could occur even if the model is apparently highly predictive. In that case, the distance of the compounds to the training set model space can be used to estimate the probability of the prediction reliability.

Generally speaking, a global model built on a large diverse data set would be expected to generate a better prediction on an unknown compounds compare to one generated on a small set of compounds. However, in some cases QSAR models built on small congeneric series can be highly useful when restricted to the chemotype in question. This is particularly true if these related molecules act via a similar mode of action so that the activity to be explained is affected by fewer factors. These so called local QSAR models are built using only a specific class of chemotypes, and have a limited domain of applicability [150, 152]. However, they are often more predictive for the subset of chemicals that they can be applied on specifically because of the fewer confounding factors contributing to the activity. Additionally, in terms of interpretation, local models can be more useful in a practical sense because it is possible to understand what the models are telling in order to obtain new molecular insights from the model. Therefore, when a novel compounds under investigation have a common structural cores, a medicinal chemist could carefully choose chemist friendly descriptors not only to get a robust model but also provide useful information on which descriptors that modulate biological activity. Local model can provide useful information to the medicinal chemist on how to improve biological activity by linking the descriptors (e.g. lipophilicity, electron donating or withdrawing properties, hydrogen bonding effects and molecular size).

The domain of applicability of QSAR models can be used to give the user a degree of confidence in the prediction as it can be shown that there is often a correlation between the query compounds and those in the training set as calculated using the model descriptors [143, 152]. It is expected that compounds that lie within this reason should be better predicted than those that lie outside, assuming of course that the model is not overfitted. The compounds from the test set, if reasonably similar to those from training set (i.e. due to selection procedure, or due to random sampling), then the model should perform well. However, if more challenging validation sets are chosen, such as unrelated compounds, or compounds prepared at a later date which typically show greater dissimilarity, the statistics are generally less favourable [143]. However, if the test chemical is far away from the training set a valid prediction cannot be expected. Once the model is established, then one can make a prediction and consider its reliability. To inform the quantity of the information available to the model, information towards a query structure can be obtained by averaging distance (e.g. Tanimoto, Euclidean, etc.) between the nearest neighbors. The reliability of the information that is in the model for a given prediction is normalized into 0 to 1 range in which 0 has the nearest distance and 1 the farthest distance. If the query compounds are in the AD of the model and the prediction is in the reliability domain, then the prediction can be concluded as valid and reliable [143].

Guidelines on the development of robust QSAR models based on the Organization of Economic Cooperation and Development (OECD) principles of validation have already been published [153]. With recent emphasis being placed on the reproducibility of models, Judson et al. [154] proposed the Good Computer Modelling Practice (GCMP) guidelines which identifies the best practice for conducting and recording modelling procedures. Although, with the availability of ample literature on the best practice in QSAR modeling [155], it is mostly aimed at those having cheminformatics/mathematical understanding of the subject. In a recently published article, Patel et al. [156] assessed the reproducibility of QSAR models pertaining to ADME predictions by scientists without expertise in QSAR. The authors reviewed 85 papers spanning 80 models with ADME related endpoints and presented a pragmatic workflow for the implementation of QSAR models with greater usability. In addition, QSAR models are able to correlate the physicochemical properties of a structure with the biological activity [157]. Hence, the QSAR models can be efficiently used for the activity prediction of unknown compounds and designing new compounds for that particular activity. However, many of the QSAR models published, are not aimed at drug design. In that regard, Kurdekar and Jadhav [158] designed an open source Python script for QSAR model building and validation using data for Matrix Metallo-Protease 13 (MMP13) inhibitors and a series of anti-malarial compounds.

### Structure-based approaches

Structure-based computational approaches generally require greater input from the model builder, resulting in a larger number of approximations being used to generate the predictive model. For example, which protein crystal structure do we choose for a particular target, how do we treat ionizable residues, are all residues flexible, what method and parameters will we use to model the results, what software program and custom parameters will we use etc. This results in a large amount of often subjective decision being made. However, while these results may change the outcome of the simulations, it is hoped that it will not impact on the overall conclusions.

For example, there are numerous protein structures that are available from multiple families via the Protein Data Bank (PDB) [159] therefore it is possible to either obtain high quality structures of the target of interest or generate homology models [160] for use in computational analysis. One of the most commonly employed technique is molecular docking, a technique that samples and scores conformations of small molecules bound to a target active site [161, 162]. Docking and scoring algorithms are employed to predict protein-bound conformation, virtual screening of large data sets and sometimes to try and estimate molecule potency.

The information gained from docking exercises can be invaluable for helping rationalize SAR and help inspire further synthetic plans. However, care should be taken as to not over interpret such models. A detailed study by Warren et al. [163] shows that although docking could successfully predict the protein-bound conformation and explore conformational space to generate corrected post as well as correctly identify molecule or chemotypes of actives from a population of decoy molecules, they are less successful in identifying the post closest to the crystal conformations using scoring functions and that no single docking protein performed well across multiple protein targets. The inconsistencies of the docking program to reproduce greater than 35% of the binding modes within 2 Å across all targets highlight the fact that experts in the loop, or additional experimental data is often needed to correctly predict binding modes.

QSAR can also be applied to 3D models of ligands that are (a) superimposed together based on a common active conformation or (b) superimposed based on how they dock within a given active site. All the complications that are pertinent to the generation of 2D QSAR models also apply to 3D QSAR. However, there are additional issues that can arise in 3D QSAR models due to the extra assumptions that must be made: (1) receptor binding is related to the biological activity (2) molecule with common structures generally bind the same way, (3) the properties that govern the observed biological response are determined by non-bonding forces, (4) the lowest energy conformation of compound is its bioactive conformation and (5) all the ligands in the study bind the same target site and have a comparable binding mode or similar mode of action [164, 165]. To perform a 3D QSAR, the chemical structures are optimized using molecular mechanics, or to a lesser extent semi-empirical and quantum mechanics to obtain a lowest conformer. These can then be overlaid on a common ligand scaffold and these coordinates are used to determine descriptors (e.g. CoMFA and CoMSIA). Chemical structures can also be superimposed using docking to a target binding site, using field based or pharmacophore-based methods. The whole process is predicated on the fact that modelled 3D structure corresponds to the active conformation. A further limitation of such models is that, compound hydrophobicity is not so well quantified, and many descriptors are produced, most of which have low variance [166].

3D models can be further expanded with additional approximations. For example, molecular dynamics simulations that are based on empirical MM parameters can be applied to simulate how molecular systems evolve over time using Newtonian mechanics. These simulations are based on rather simple molecular methods (e.g. AMBER, CHARMM parameters, etc.) which is necessary to obtain sufficient conformational sampling. Nevertheless, computational sampling could be sacrificed if the user wanted to use more accurate quantum mechanical methods, starting with semi empirical treatments such as AM1 or PM3, to ab initio methods such as Hartree Fock (HF) to methods that take into account electronic methods such as DFT (e.g. B3LYP and M0X series) [167]. A further advantage of the latter methods is that they do not require bespoke generation of parameters for each molecule. However, the massive overhead in computation means they are rarely used for protein simulations. Alternative methods to get around the flaws of both methods include hybrid quantum mechanical/molecular mechanical methods. In these methods the active site region that contains the substrate/inhibitor and the main residues it interacts with are defined using QM, and the remaining protein MM. This method makes it possible to perform more accurate evaluation of the energetics while also providing a means to perform molecular dynamics over acceptable time frames [168–172]. Despite these advances, a suitable balance between methods of sufficient accuracy, and sampling of sufficient time eludes us.

### Systems-based approaches

Systems-based drug discovery aims takes a holistic view of the genome, proteome and their specific interactions amongst one another and how chemicals may postively or negatively modulate their action [173, 174]. Particularly, this encompasses the understanding of the underpinning details of biochemical pathways in which the interplay of gene, proteins, carbohydrates, lipids and chemicals sustain the molecular logic of life. As there are more than 30,000 genes that may subsequently translate to proteins via complex gene expression feedback loop, therefore such vast amounts of data requires the utilization of computers for extracting key insights. Systems biology take a broader overlook of biological systems as oppose to the convention reductionist approach. The field pieces together disparate information from various omics disciplines to produce a unified analysis of the data.

In the context of drug discovery, systems pharmacology (i.e. also termed “network pharmacology”) makes it possible to perform drug repositioning [175, 176] in which known FDA-approved drugs that were originally designed to treat disease A (i.e. original indication) can be repurposed or repositioned to treat other diseases (i.e. new indication). This is made possible owing to the concept of polypharmacology that essentially relies on the concept of molecular similarity whereby similar target proteins are assumed to also share similar binding characteristics to compounds [177]. For instance, the nelfinavir (i.e. an HIV-1 protease inhibitor) has been demonstrated to exert promising anti-cancer activities against a wide range of cancer types [178]. Aside from network pharmacology proteochemometric modeling is systems-based approach that has also been demonstrated to facilitate drug repositioning [179, 180].

Hereafter, we examine the ongoing work in the effort to establish reproducibility of systems biology models. The COmputational Modeling in BIology NEtwork (COMBINE) is an initiative that has been set up in 2010 to coordinate the development of various community standards and formats pertaining to the development of systems biology models. Two independent articles published in the *IEEE Transactions on Biomedical Engineering* examines this topic in which Waltemath and Wolkenhauer [181] focused on how initiatives, standards and software tools supports the reproducibility of simulation studies while Medley et al. [182] formulated a set of guidelines for building reproducible systems biology models. Moreover, Waltemath et al. [183] outlined the necessary steps needed to facilitate the production of reproducible models in the systems biology setting by exemplifying a number of computational models pertaining to the cell cycle as obtained from the BioModels database [184]. The authors summarized that in order for models to be reproducible, they should be (1) encoded in standard formats (e.g. XML, SBML, CellML, etc.), (2) the meta-information should be provided to support the understanding of the model’s intention, (3) associated simulation experiments should be encoded in standard formats and (4) all information must be made available through open repositories.

Kirouac et al. [185] investigated the reproducibility of quantitative systems pharmacology (QSP) by analyzing 18 QSP models published in the *CPT: Pharmacometrics and Systems Pharmacology* journal. Owing to the heterogeneity of the platform used in the 18 models, 12 were selected for further analysis (i.e. coded in R, PK-Sim/MoBi, and MATLAB) and only 4 were found to be readily executable from a single run script. From there, the authors initiated points for discussion on how to establish best practices for QSP model reproducibility. Notable points raised includes: (1) suggesting the provision of a single run script to allow interested users to easily perform the simulation, (2) journals should provide recommendations on the sharing of code and data, (3) provide sufficient details on the setup of the simulation model, (4) provide models in open source, standardized format, (5) provide details on the computation environment (e.g. software version, parameter details, etc.).

Watanabe et al. [186] discussed the challenges of disease model reproducibility that had predominantly relied on periodically evolving loose guidelines as opposed to well-defined machine-readable standards. Thus, the authors investigated the utility of Systems Biology Markup Language (SBML) [187] in the development of disease models as compared to other associated languages including Pharmacometric Markup Langugae (PharmML)[188] and MIcro Simulation Tool (MIST) [189]. Results indicated the robustness of SBML for model reproducibility and as the authors pointed out, there exists substantial adoption of SBML where most are being deposited to the BioModels repository [184].

In addition to aforementioned markup languages for facilitating the exchange of models, there also exists other languages as well such as CellML[190] , Simulation Experiment Description Markup Language (SED-ML) and Systems Biology Graphical Notation (SBGN). In the presence of these various markup languages, the research group of Sauro [191, 192] proposed the Tellurium platform as an integrated environment (i.e. models, Python code and documentation; similar to a Jupyter notebook) that is designed for model building, analysis, simulation and reproducibility in systems biology while facilitating the use of multiple, heterogeneous libraries, plugins as well as specialized modules/methods. Similarly, BioUML[193] is a web-based, integrated platform that facilitates the analysis of omics data in the context of systems biology. Furthermore, the extensive collection of the plug-in architecture (i.e. comprising more than 300 data analytic methodologies coupled with its ability to integrate with the Galaxy and R/Bioconductor platforms) positions BioUML as a prominent platform for building systems biology models. Moreover, a workflow engine integrated into the BioUML helps to support the concept of reproducible research as new input data can be plugged into the already existing model pipeline. Additionally, Drawert et al. [194] developed MOLNs as a cloud appliance that entails setting up, starting and managing a virtual platform for scalable, distributed computational experiments using (spatial) stochastic simulation software (e.g. PyURDME).

## Computational issues on model development and deployment

There are two main issues facing the computational scientist or model developer in drug development: computational processability and scalability. Irregardless of where computation is performed (i.e. on a laptop, a server, a data center or a cloud infrastructure) in order to achieve the reproducibility in sufficient details, it is crucial that tools for structuring and managing these processes are implemented and exploited as drug research pertains to many activities involving various data types of different sizes and formats. In a typical computational drug discovery project, it becomes very difficult to keep track of tools and parameters that were used, the different versions of data as well as the manual gluing of results together into the final tables and figures that are presented in a scientific manuscript. Proper data management becomes a key necessity. Challenges of data management in the big data era have previously been discussed [195] and practical suggestions on how to structure data in computational analysis projects have also been proposed [196]. Furthermore, as soon as a data set increases in size above a few tens or hundreds of gigabytes, or when the amount of data needed to be kept in RAM becomes larger than a few gigabytes, it often becomes infeasible to perform computations on the user’s own local laptop, and therefore scaling up the computation on a larger computing infrastructure becomes an inevitable need. In this section, we discuss the most common approaches for resolving these challenges.

### Scientific workflow management systems

For reproducible research, an important capability is to be able to re-run and validate a complete analysis pipeline in an automated fashion. While this can, to some extent, be done by scripting, scripted pipelines can easily become brittle and complex to manage and modify due to their low-level nature. As the user is forced to take care of all the low-level details of data management and program execution, even simple changes in the workflow can require a substantial mental effort in order not to introduce subtle errors. Also, tracking intermediate output from an intermittent process of the pipeline can be difficult in order to determine which process causes the failure. Optimizing which step to be rerun instead of restarting the whole pipeline is an important question in the context of large-scale analyses. These problems are at the core of what scientific workflow systems aim to solve, and thereby contributing to making computation research more reproducible.

Scientific Workflow Management Systems (WMS) provide a number of added benefits to computational pipelines that help in creating reproducible, transparent computations. Firstly they allow the user to construct the pipeline using a more high-level, abstract description than plain scripts, hiding away low level technical details of exactly how data is managed and programs are executed. The user typically only needs to specify how the computational steps depend on each other, and which parameters to feed them with. The WMS takes care of low-level details such as scheduling the concrete invocations of the workflow steps in the right order with the right parameters, passing on data between processing steps, separating unfinished and finished files (in what is often called atomic writes), logging, producing audit reports and more.

The more high level description of workflows in WMS, primarily consisting of task and data dependencies, makes it easier to follow the logic of the core computation making up the pipeline. It also makes the workflows easier to change as one typically needs to change the workflow code in far fewer places than in scripts, because of the lower amount of details specified in the high-level description.

In summary, WMS benefit reproducible computations by (1) making automation of multi-step computations easier to create and more robust and easy to change (2) providing more transparency to what the pipeline does through its more high-level workflow description and better reporting and visualization facilities, and (3) by providing a more reliable mechanism for separating unfinished and completed outputs from the workflow.

**Fig. 3:**
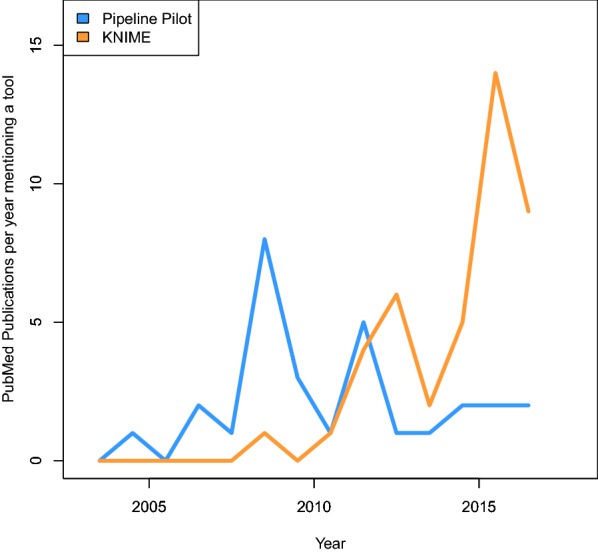
Number of articles on PubMed, mentioning *“Pipeline Pilot”* or *“KNIME”* in their title or abstract from 2003 to 2017

The most common WMS used in drug discovery over the last few decades have been the proprietary Pipeline Pilot software [197] and the open source KNIME workbench [198], which also has proprietary extensions. Over the years, the relative use of KNIME appears to have increased. This is illustrated by the distribution of articles available on PubMed which mention *“Pipeline Pilot”* or *“KNIME”* in their title or abstract (Fig. 3). It should be noted that a PubMed search might not provide the full picture of the usage of these software inside for example pharmaceutical industry, since PubMed only indexes public, peer-reviewed articles, the production of which is not the primary concern for most pharmaceutical companies. A part of the increased number of papers mentioning KNIME might also be due to the generic nature of the platform, as KNIME is not focusing only on drug discovery, but also has support for more general data analysis tools, as well as tools specific to other fields even outside of the biomedical domain.

Pipeline Pilot has been used in several studies, including to design screening libraries [197], for high-content screening [198], and for compound design [199]. KNIME has been used, for example, for virtual screening [200], target identification [201]; more in-depth coverage of applications are provided elsewhere [202].

In addition to Pipeline Pilot and KNIME, there has been some use of the Taverna and Galaxy platforms too. Taverna, which has been widely used in the wider bioinformatics field in the past, has functionality relevant to drug discovery through the CDK-Taverna project [203], which integrates the JVM-based Chemistry Development Kit [204, 205]. The immensely popular web based Galaxy platform [49–51] has the ChemicalToolBoX, which is a suite of more than 30 tools for chemistry and cheminformatics integrated [206].

A recent trend among many more recent workflow tools popular in bioinformatics, is that the main mode of interaction with the user is increasingly often purely text-based. Prominent examples of this trends include tools like Nextflow [207], Snakemake [208], Ruffus [209], BPipe [210], Cuneiform [211] and Luigi [212]. Discussions with users of workflow tools reveals that this focus has a lot to do with the easier integration of workflows into HPC and cloud computing environments as well as easier version control when all workflows are stored as plain text files rather than as configurations in a GUI software. Keeping track of all changes and versions to workflows in version control is identified as one key component in achieving reproducibility in computational biology [213, 214].

Among these newer text-based tools, Luigi has found some use in drug discovery. The fact that Luigi is implemented as a Python library, enables it to seamlessly integrate with python based client programming libraries such as the ChEMBL client library [215]. By not requiring a GUI, Luigi is also easier to integrate and run in an HPC environment, interacting with resource managers such as SLURM. This was recently done in a study on the effects on dataset and model sizes on the predictive performance of toxicity models [216]. SciLuigi [217] is a wrapper library around Luigi, designed specifically to make workflow motifs common in drug discovery easier to model with Luigi. An example of of such motifs are machine learning pipelines containing cross-validation of trained models, nested with parameter sweeps. SciLuigi also includes built-in support for the SLURM HPC resource manager [218].

Another trend in the wider field of computational biology is increasing adoption of support for tool-agnostic, interoperable workflow description formats such as the Common Workflow Language [219] or Workflow Description Language [220]. Such tool-agnostic formats promise to make it easier to share workflows with other users, who might prefer or even be restricted to, other tools and infrastructures, and can thereby make reproduction of computational studies easier. Use of such interoperable formats has yet to see widespread use within drug discovery, but presents a promising direction for increasing the reproducibility of computational studies in the field. By being a textual representation of workflows, they may also provide an excellent way for GUI-centric workflow systems to provide a representation of its workflows that fits in easily with popular version control systems like Git.

### Large-scale integrative computational infrastructure

#### High performance computing (HPC) clusters

The traditional way of scaling up scientific computing workloads has been by using high performance clusters. These have in the last couple of decades typically consisted of so called Beowulf clusters, meaning clusters composed of relatively *“normal”* computers, running a common operating system such as Linux, and connected through a high performance network. These compute nodes typically mainly only differ from normal computers by possibly having more compute cores and/or random access memory (RAM). Workloads on HPC clusters can either run within one node, much like any other program, or use a technology such as Message Passing Interface (MPI) to run a computation by running the program on multiple nodes, where the multiple instances communicate with each other via MPI. The latter is a common scenario in physics, but is not widespread for computations in the biomedical field.

Despite of the recent trend towards cloud computing environments, HPC still remains a common option especially for academic computing because of the relatively low cost per CPU hour. On the other hand, HPC environments typically do not allow the same level of flexibility and user control as cloud environments, because of tighter security requirements, and various policies induced by local system administrators. For example, it is typically out of question to get root privileges on a HPC compute node, or to install your own virtual machine, where you could get root privileges. This means users sometimes need to compile and/or install the required software by hand, if the right version of the software they need is not already available on the cluster. There are some recent trends to meet the need for software packaged into container, most notably through the Singularity project, which allows users to run a type of container without root privileges.

#### Cloud computing and virtualization

Cloud computing offers computational infrastructure, platforms, and services on-demand, and it will have a profound impact on how computational drug discovery is carried out [221, 222]. For pharmaceutical companies, on short term perhaps the highest impact is the on-demand availability of computational infrastructure, relieving them of the burden to manage an in-house computing center. But in the longer run, platforms-as-a-service supporting drug discovery has the potential to dramatically change the way computer-aided drug discovery is carried out, for example, accelerate processes [223] and scaling up analyses [224], but also at the same time drastically improve reproducibility.

#### Virtual machines

Some software tools and workflows/pipelines can be complex to move between systems, even if they are open source and all data is publicly available. For example, when installing the same software on different systems, there will always be different versions in some dependent packages and different optimization flags for compilations etc. that could affect the execution of software and lead to different results in analysis [207]. One way of addressing this problem is by using virtual resources. A virtual machine (VM) is an emulation of a computer system that provides functionality of a physical computer, with a complete operating system that runs within a managed *“virtual”* environment without direct connection to the underlying *“host”* computer. Virtual machines can be packaged as a virtual machine image (VMI or simply *“image”*) that can be transported between systems and launched on demand. In science, researchers can take a “snapshot” of their entire working environment including software, data, scripts etc that can be shared or published, and cited in publications to greatly improve reproducibility [225, 226].

VMs have been used in several drug discovery projects. For example, Jaghoori et al. [227] described how AutoDock Vina can be used for virtual screening using a virtual machine. McGuire et al. [228] developed 3d-e-Chem-VM, a virtual machine for structural cheminformatics research. Lampa et al. [217] provides a complete analysis using predictive modeling in drug discovery that is shared as a virtual machine image. Lilly has developed their Open Innovation Drug Discovery platform [229] where participating investigators get access to tools and predictions by Lilly software and data via a virtual machine where they can, for example, submit compounds for in silico evaluation. The widely used ChEMBL database makes the data and tools available as a virtual machine via the myChEMBL package [230]. Virtual machines are also a necessity for Big Data frameworks in drug discovery, for example, implementing docking on Hadoop [231] and Apache Spark [232]. VMs can also be useful for providing student environments for educational courses, such as is done for the course Pharmaceutical Bioinformatics at Uppsala University [233]. There are several places to deposit virtual machines, for example, the BioImg.org website [234] is a catalog dedicated to housing virtual machine images pertaining to life science research. Further, VMIs can be shared within several public cloud providers (see Table 1).

**Table 1:** List of the largest public cloud infrastructure service providers

Service provider	URL
Amazon Web Service	http://aws.amazon.com/
Microsoft’s Azure	http://azure.com/
Google Cloud Platform	http://cloud.google.com/
IBM’s SoftLayer	http://www.softlayer.com/
Alibaba Cloud	https://www.alibabacloud.com/

Service providers are ordered according to market share [284]

#### Containers

**Fig. 4:**
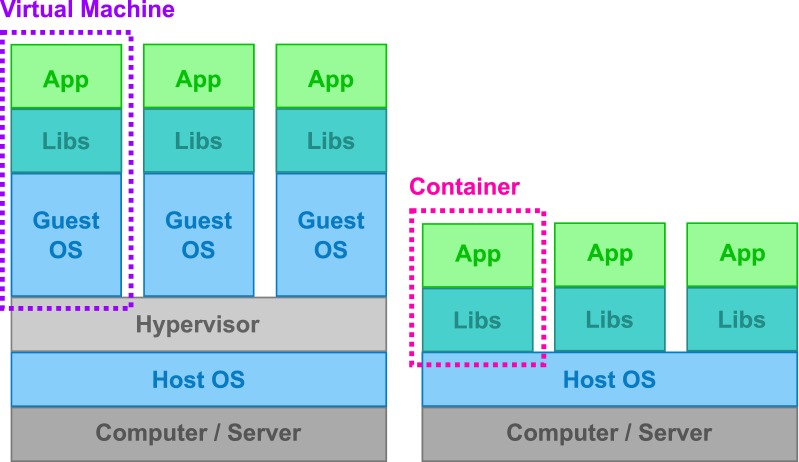
Schematic comparison of virtual machines and containers. Virtual machines run on a Hypervisor and contains their own Guest Operating System. In contrast, Containers provide a layer of isolation that share the Host Operating System kernel and are hence smaller and faster to instantiate than virtual machines

A drawback of VMs to support computational reproducibility is that VMIs, with all software and raw data for an analysis available, tend to become rather large (i.e. in the order of several gigabytes). Software containers, or simply ‘containers’, are similar to virtual machines that they isolate software from its surroundings, but a container is smaller and do not contain the entire operating system; in fact, several containers can share the same operating system kernel making them more lightweight and use much less resources than virtual machines (Fig. 4). Containers can hence aid reproducible research in a way similar to virtual machines, in that they produce the same output irregardless of the system or environment it is executed on [226, 235, 236]. The most widely used containerization technology is Docker [70], but Singularity [237] and uDocker [238] are compelling alternatives that can run without root privileges and hence are more useful in shared high-performance computing facilities.

It is quite straightforward to containerize tools, and due to the portability it has become popular to ship tools for workflow environments such as Pipeline Pilot and KNIME [239]. However, containers in drug discovery is a relatively recent technology and not many published studies are available. Suhartanto et al. [240] presents a study for shifting from virtual machines to Docker containers for cloud-based drug discovery projects. The pharmaceutical company GSK describes in a presentation at DockerCon 2017 how they are able to accelerate science with Docker [241]. Altae-Tran et al. [242] applies Deep neural networks, available as a containerized version of their package DeepChem. Further, container technology is empowering e-infrastructures relevant for drug discovery, such as the OpenRiskNet project [243].

There are several repositories for containers, with Docker Hub being perhaps the most widely used. However, catalog services and standardization initiatives relevant for life science research also exist, with Bioboxes [244] and BioContainers [71] as two prominent examples. With the growing popularity of containers, it is very likely that we will see more virtualized tools, environments and studies become available using this technology in the future which will contribute to reproducible research.

#### Model deployment

**Fig. 5:**
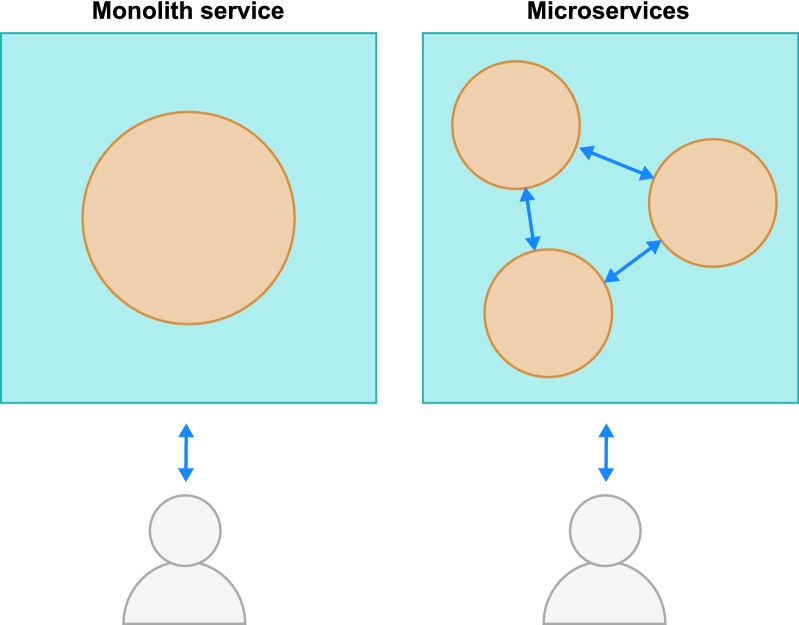
A comparison between monolith services and microservices. In traditional services (left), each service consists of a monolithic implementation that encapsulates all necessary components under a single interface. In contrast, a Microservice-based implementation (right) has the individual components that make up an exposed service running independently, making it easier to scale parts of the service if needed as well as offering the benefit of reusing sub-components in other settings

Deploying a model in this context refers to installing it in a way so that it becomes accessible to oneself or others (Fig. 5). A model could, for example, be deployed on a laptop, a server on an internal network, on a private cloud for a selected group of people, or as a public service. Traditional model deployment as a service has commonly been done as a Web service available over a network, such as Internet. The service can then be accessed either via an HTML page that calls an application server that delivers results from the model, or via a Web API that can be consumed programmatically by software applications. There are some limitations of this simple model:

1. The service provider needs to maintain the service and the computer it runs on. If the service goes down, it should be restarted. Security patches must be applied. Hardware must be upgraded and replaced over time. This places a considerable burden on the service provider.
2. Whenever an update is made to the service, the version and possibly API will have to be changed. In order to sustain reproducibility, this soon leads to the maintenance of multiple versions on the same service.
3. If the service is resource-demanding, it can be expensive to offer it as a free service.

These problems have limited the use of models deployed as services, apart from in-house services at companies with adequate system and service support.

Owing to the inherent complexities involved with setting up and maintaining fault-tolerant and scalable services, provisioning model services as virtual machines and containers has attracted a lot of interest [245]. Here it both becomes easier to publish a model online on, for instance, a cloud provider that eliminates the need to buy and maintain computational hardware, but also to enable users to instantiate the service on their own computational infrastructure. With proper versioning of services available (e.g. Docker containers) the end users can download and instantiate explicit versions of the model and ensure a reproducible component of an analysis. The problem becomes more how input and output data is structured, and there is a need for the community to develop and agree upon such standards for data, metadata including ontologies and vocabularies, and discoverability in order to promote interoperability among models deployed as services.

## Use case scenarios for streamlining the computational drug discovery protocol

### Workflows for computational drug discovery

In a real-life scenario, a typical research project in computational drug discovery involves the use of several software, programs and tools that spans from reading input files, data pre-processing, one or more rounds of computation and post-analyses. This would likely involve pre-processing and connecting the outputs of one software or tool as input to another software or tool. Such task may be a troublesome endeavor that may require manual pre-processing of the output and input files. Such issue may potentially be solved if software or tool developers also consider the practical use case scenario pertaining to the interoperability of input/output files for various software and tools.

In cheminformatics research, there are efforts to establish standardized formats and repositories for QSAR models and data. In order to foster reproducible QSAR, exchange formats for data, models, and parameters are needed. QSAR-ML is an XML-based exchange format aimed at promoting interoperable and reproducible QSAR data sets, building on an open and extensible descriptor ontology [246]. The QSAR DataBank (QsarDB) [247, 248] is a repository that aims towards making QSAR modelling transparent, reproducible and accessible via a custom file format and services.The QSAR Model Reporting Format (QMRF) is a harmonised template for summarising and reporting key information on QSAR models, including the results of any validation studies. The information is structured according to the OECD validation principles and is used by the JRC QSAR Model Database [249]. QMRF version 3.0.0 has been updated within the context of the eNanoMapper project [250].

There are also additional general exchange formats for machine learning that are relevant for predictive models in cheminformatics. Predictive Model Markup Language (PMML) [251] is an XML-based predictive model interchange format that also includes data transformations (pre- and post-processing). PMML is sustained by the Data Mining Group [252]. The latest version of QMRF has basic support for PMML. The KNIME workflow software also has support for PMML [253] and the QSAR DataBank (QsarDB) [247, 248] also supports the exporting of models in the PMML data format. A more recent format is the Open Neural Network Exchange (ONNX) that provides an open source format for AI models (i.e. both deep learning and traditional machine learning) [254]. So far there is no reported usage within cheminformatics but the increasing interest in deep learning makes this a relevant candidate for future exchange of models.

In regards to QSAR workflows, there have been considerable efforts directed at this important endeavor that typically entails the utilization of several programs and tools and a series of intricate data pre-processing, model building and analyses (Table 2). Stålring et al. [255] presented an open source machine learning application called AZOrange that allows QSAR model building in a graphical programming environment. Dixon et al. [256] proposed the AutoQSAR as an automated machine learning tool for QSAR modeling using best practice guidelines that was validated on six biological end-points. Nantasenamat et al. [257] reported the development of an automated data mining software for QSAR modeling called AutoWeka that is based on the machine learning software Weka [258]. Kausar and Falcao [259] presents an automated framework based on KNIME for QSAR modeling entailing data pre-processing, model building and validation. Dong et al. [260] introduced an online platform for QSAR modeling known as ChemSAR that is capable of handling chemical structures, computing molecular descriptors, model building as well as producing result plots. Tsiliki et al. [261] proposed an R package known as RRegrs for building multiple regression models using a pre-configured and customizable workflow. Murrell et al. [262] introduced an R package known as the Chemically Aware Model Builder (camb) that continues where the general-purpose R package RRegrs left off which is the capacity to handle chemical structures (i.e. desalting and tautomerizing chemical structures as well as computing molecular descriptors). Shamsara [263] presents yet another R package for QSAR modeling called Ezqsar.

**Table 2:** List of software and packages that implements an automated QSAR modeling workflow

Software/tool	Description	URL	Refs.
Standalone and online applications			
AZOrange	Graphical programming environment based on the Python package “Orange” for performing QSAR modeling workflow	https://github.com/AZcompTox/AZOrange/	[255]
AutoQSAR	Automated machine learning tool for QSAR modeling using best practice guidelines	https://www.schrodinger.com/autoqsar/	[256]
AutoWeka	Automated data mining software for QSAR modeling based on the machine learning software Weka	https://www.mt.mahidol.ac.th/autoweka/	[257]
ChemSAR	Online platform for QSAR modeling that is capable of handling chemical structures, computing molecular descriptors, model building as well as producing result plots	http://chemsar.scbdd.com/	[260]
Tools implemented in R language			
camb	R package that is capable of handling chemical structures, compute descriptors and build QSAR models	https://github.com/cambDI/camb/	[262]
Ezqsar	R package for building QSAR models	https://github.com/enanomapper/RRegrs/	[263]
RRegrs	R package for building multiple regression models using pre-configured and customizable workflow	https://github.com/enanomapper/RRegrs/	[261]

Additionally, easy to follow/share pipelines for drug discovery is largely facilitated by the open source nature of the above mentioned cheminformatics and structural biology workflows. Recently, one of us published a book chapter on the construction of reproducible QSAR models [264] in which key factors influencing the reproducibility of QSAR models (i.e. data set, chemical representation, descriptors used, model’s parameters/details, predicted endpoint values and data splits) and guidelines on using Jupyter notebook for building reproducible QSAR models are provided. As such, Jupyter notebook is a popular platform in which these workflows are coded, owing to its intuitive blend of code and documentation. Particularly, the ten simple rules for best practice in documenting cheminformatics research using the Jupyter notebook is a useful and timely guideline [265]. These documentations can also be found on GitHub, where a number of researchers share the code to their project’s workflow. A selected group of such researchers and the specific area of computational drug discovery research (e.g. ligand-, structure- and/or systems-based) are summarized in Table 3. From this table, we can see that Greg Landrum [266] has shared Jupyter notebooks pertaining to the use of the RDKit module [267] in the context of ligand-based drug discovery on his personal GitHub as well as contributing to the RDKit GitHub [268]). In addition, the OpenEye Python Cookbook [269] is a collection of practical solutions to ligand- and structure-based drug discovery research (i.e. combinatorial library generation, substructure search as well as ligand and protein-ligand structure visualization). Furthermore, myChEMBL [230] is an open source virtual machine that combines bioactivity data from ChEMBL with the latest RDKit [267] cheminformatics libraries to sustain a self-contained and user-friendly interface. Putting a new twist to conventional Jupyter notebook, Squonk [270] is a web-based workflow tool based on Jupyter notebook for computational chemistry and cheminformatics for processes encompassing ligand- (i.e. combinatorial library generation, 3D conformer generation, prediction of metabolism and toxicology, molecular property prediction, data visualization and analysis as well as clustering and diversity analysis) and structure-based virtual screening (i.e. scoring active site conformation of compounds).

**Table 3:** List of selected GitHub URLs of researchers working in the domain of computational drug discovery

Researcher’s name	GitHub URL	Ligand-based	Structure-based	Systems-based
Andrea Volkamer	https://github.com/volkamerlab/	✔	✔	
Chanin Nantasenamat	https://github.com/chaninlab/	✔		✔
	https://github.com/chaninn/	✔		
Egon Willighagen	https://github.com/egonw/	✔		
George Papadatos	https://github.com/madgpap/	✔		
Greg Landrum	https://github.com/greglandrum/	✔		
Jan H. Jansen	https://github.com/jensengroup/	✔	✔	
John Chodera	https://github.com/choderalab/	✔	✔	
Ola Spjuth	https://github.com/olas/	✔		
Rajarshi Guha	https://github.com/rajarshi/	✔		
Samo Turk	https://github.com/samoturk/	✔		

Aside from the research aspect, educational code-based tutorials on computational drug discovery has been initiated using the Java-based Chemistry Development Kit (CDK) [204, 205, 271] as implemented by the Teach-Discover-Treat (TDT) initiative [272]. This resulted in the development of Python-based tutorials pertaining to the virtual screening workflow to identify malarial drugs [273, 274]. Furthermore, the recently launched TeachOpenCADD platform [275] complements the already available resources by providing students and researchers who are new to computational drug discovery and/or programming with step-by-step *talktorials* that cover both ligand- and structure-based approaches using Python-based open source packages in interactive Jupyter notebooks [276].

Similarly, a software platform in structural bioinformatics known as Biskit [277] links several common tasks in molecular simulation (i.e. each task is a modular object) into a complex workflow that allows streamlined execution of these tasks in a concerted manner. Particularly, researchers can pre-process and analyze macromolecular structures, protein complexes and molecular dynamics trajectories via automated workflow making use of established programs like Xplor, Amber, Hex, DSSP, Fold-X, T-Coffee, TMAlign and Modeller.

In summary, the use of these computational workflows (i.e. that have been tailored to rigorously handle the specific task of interest such as building QSAR models, pre-processing protein structures for molecular simulations, etc.) further helps to ensure the computational reproducibility of the procedures as they have been pre-configured to do so.

### Web servers for computational drug discovery

In recent years, the advent of web technologies and the convenience with which users can make use of the functionalities of web-based applications has led to the development of a wide range of web tools and applications in the realm of bioinformatics and cheminformatics for aiding drug discovery efforts (Table 4). The obvious advantage of these web applications is that there is no hassle for installing and maintaining their own computational infrastructure for performing such tasks. The extent of these tools can fall into any one or more of the following tasks: data curation, pre-processing, prediction and analysis. Moreover, another advantage borne from this is the fact that such web applications support reproducibility in that the underlying protocol being performed by the tool is iteratively executed in the same manner regardless of the number of times it is initiated. In efforts to facilitate easier dissemination of bioinformatic applications as web server, Daniluk et al. [278] introduced the WeBIAS platform, which is a self-contained solution that helps to make command-line programs accessible via web forms. In spite of its advantages and potential utility for the scientific community, the only downside of web databases and applications is the possibility that they may be discontinued at any time. In fact, a recent review explores this issue in which Ősz et al. [279] investigated 3649 web-based services published between 1994 and 2017 and discovered that one-third of these web-based services went out of service. Such discontinued support of web tools and resources poses a great impediment to research reproducibility.

**Table 4:** List of selected web applications for handling various bioinformatic and cheminformatic tasks belonging to either ligand-based or structure-based drug design approach

Web servers	Description	URL	Refs.
Ligand-based drug design			
BioTriangle	Compute descriptors for compounds, protein, DNA and their interaction cross-terms	http://biotriangle.scbdd.com/	[285]
ChemDes	Computes 3679 molecular descriptors and 59 fingerprint types for compounds	http://www.scbdd.com/chemdes/	[286]
ChemBench	Enables QSAR model building via pre-defined workflow	http://chembench.mml.unc.edu/	[287]
OCHEM	Online platform providing storage for QSAR data and workflow for model building	http://www.ochem.eu/	[288]
PUMA	Performs analysis and visualization of chemical diversity	https://www.difacquim.com/d-tools/	[289]
Structure-based drug design			
HADDOCK	Performs information-driven docking of biomolecular complexes (e.g. DNA, proteins, peptides, etc.)	http://haddock.science.uu.nl/services/HADDOCK2.2/	[290]
FlexServ	Performs coarse-grained determination of protein dynamics	http://mmb.pcb.ub.es/FlexServ/	[291]
MDWeb	Provides standard protocol for preparing structures, run standard molecular dynamics simulations and analyze trajectories	http://mmb.irbbarcelona.org/MDWeb/	[292]
PoseView	Displays simple molecular interaction diagram of protein-ligand complexes	http://www.zbh.uni-hamburg.de/poseview	[293]
SwissModel	Predicts protein structures via template-based homology	https://swissmodel.expasy.org/	[294]

In recent years, the availability of Shiny [280] and Dash [281] packages for the R and Python programming environment, respectively, has greatly lowered the technical barrier to web development for typical R and Python users by facilitating the rapid prototyping of computational workflows as a sharable web-based application. Plotly [282] represents a robust tool for producing interactive data visualization that can be collaboratively shared to colleagues. Graphs and dashboards can be made with no coding and is thus appealing to the non-technical users while the available Plotly packages for various platforms (e.g. R, Python, Javascript and React) is equally appealing to technical users as well.

## Conclusion

The dawn of the big data era in drug discovery is made possible by technological advancements in the various omics disciplines. Such big data brings with it great opportunities for advancing life sciences while at the same time bringing several potential problems pertaining to the reliability and reproducibility of generated results. In efforts to steer clear of the potential pitfalls that may be lurking ahead, it is of great importance to grasp the current state-of-the-art of research reproducibility in computational drug discovery as to ensure that the underlying work is of high quality and that it is capable of withstanding reproduction of the described methodology by external research group. A wide range of resources and tools are available for embarking on the journey towards reproducibility in computational drug discovery projects, which has been explored in this review article. The growing culture of sharing the underlying data and codes published in research articles pertaining to computational drug discovery is anticipated to drive the field forward as new and useful knowledge base can gradually be built on top of its predecessors thereby creating a snowball effect. In recent years, policies imposed by granting agencies and publishers are in favor of data and code sharing, which are further facilitated by third-party platforms (e.g. Authorea, Code Ocean, Jupyter notebook, Manuscripts.io, etc.) that further enhances reproducibility in which manuscripts and codes that are shared on the web are no longer static files waiting to be downloaded but are *“living”* codes and documents that can dynamically be edited and executed in real-time.

In summary, we have attempted to detail the diverse range of issues faced by the predictive modelling community in its role to develop and deploy efficient and reliable computational tools for drug discovery. From examples presented herein, it is clear that close interaction between frontline drug discovery scientists, the intermediate data modellers, and back office computer scientists and administrators. The challenge that each of these groups faces are quite different in nature and thus there needs to be improved understanding of these issues and a common vocabulary in order to maximize their impact. This is no small task, given the breadth of the fields involved. We note that it is of critical importance that data modellers, tool developers and administrators do not lose sight of the fact that tools must be developed for use by front line scientists in day-to-day, dynamic environment. This dynamic nature may lead to a degree of conflict with best practices espoused by the data science community (i.e. due to ever changing needs).

With this in mind, it is necessary to understand that certain solutions are preferable to the developer community and may not be considered optimal to model developers. For example, custom models using user-derived descriptors (i.e. experimental data or non-standard 3D computational models) may be desirable, but difficult to incorporate rapidly into QSAR models in a short period of time. Alternatively, predictive models that deliver lower overall predictive performance, but greater interpretability, may be preferred in some cases. The latter model types might not appear in automated solutions in now common modelling workflows as selection conditions are generally driven by statistical considerations rather than needs of the end user.

Open source promotes transparency in implementations and allows for easy access to validate analysis. When working with data and modeling, it is often difficult to keep track of tools and parameters used in the analysis. Workflow systems can aid in this and are gaining momentum in drug discovery. They contribute to more robust multi-step computations, transparency, provenance and ease of reproducibility. There is also an increased push for interoperability and standardization of workflow specifications with projects like Common Workflow Language.

With growing data sizes, the use of shared or public computing infrastructures (HPC/Cloud) is necessary and therefore adds another level of complexity for computational reproducibility. In order for all tools used for data analysis to be portable between systems, technologies such as virtual machines and software containers are widely used. When connecting containers and virtual machines with workflow systems, a high level of automation can be achieved, and through that improved reproducibility. Virtual infrastructure and containers also facilitate more reliable and replicable services, for instance, for deploying models as services over the network.

## Data Availability

Not applicable.
